# Comparative GC–MS Characterization and Antimicrobial and Antioxidant Activities of Essential Oils from Two Chemotypes of *Matricaria pubescens*

**DOI:** 10.3390/cimb48040363

**Published:** 2026-03-31

**Authors:** Elhasnaoui Abdelhadi, Janah Iman, Ait Tastift Maroua, Ouhaddou Soukaina, Sellam Khalid, El-Haidani Ahmed, Lahrach Nadia

**Affiliations:** 1 Ethnopharmacology and Pharmacognosy Team, Faculty of Sciences and Technics Errachidia, Moulay Ismaïl University of Meknes, 509 Boutalamine, Errachidia 52000, Morocco; 2 Laboratory of Agro-Food, Biotechnologies and Valorization of Plant Bioresources (AGROBIOVAL), Faculty of Science Semlalia, Cadi Ayyad University, Marrakesh 40000, Morocco; 3 Laboratory of Excellence in Agrobiotechnology and Bioengineering, AgroBiotech Center, CNRST-Certified Research Unit (URL05-CNRST), Plant Resources Protection and Enhancement Team, Cadi Ayyad University, Marrakech 40000, Morocco; 4 Biology, Environment and Health Team, Faculty of Sciences and Technology Errachidia, Moulay Ismaïl University of Meknes, 509 Boutalamine, Errachidia 52000, Morocco

**Keywords:** antibiotic resistance, antimicrobial activity, antioxidant activity, medicinal and aromatic plants, essential oil, *Matricaria pubescens*, southeastern Morocco

## Abstract

Amid the accelerating spread of antibiotic resistance, medicinal and aromatic plants stand out as powerful natural reservoirs of bioactive compounds, offering innovative prospects for next-generation antimicrobial therapies. To explore its therapeutic potential, this study evaluated the antimicrobial and antioxidant activities of *Matricaria pubescens* from Southeastern Morocco, supported by a thorough chemical profiling of its essential oils. The oils were obtained by steam distillation and analyzed using gas chromatography–mass spectrometry (GC–MS). The results revealed two distinct chemotypes, with isochrysanthemic ethyl ester (32.7%) as the dominant compound in chemotype EO1 and α-ocimene (19.62%) as the major constituent in chemotype EO2. Antioxidant activities were assessed using DPPH, ABTS, and reducing power assays, while antimicrobial activities were evaluated against bacteria, fungi, and yeasts using both disc diffusion and broth microdilution methods. Both oils exhibited notable antioxidant activities. Significant antimicrobial effects were observed, with *Bacillus subtilis*, *Escherichia coli*, and *Staphylococcus aureus* being the most sensitive strains, whereas *Pseudomonas aeruginosa* exhibited the highest resistance among all tested microorganisms, with the lowest MIC recorded for *B. subtilis* (0.612 mg/mL). These findings emphasize that *M. pubescens* could serve as a valuable source of biologically active compounds, particularly in the development of agents to combat microbial resistance, and further support its potential applications in pharmaceutical, cosmetic, and food industries.

## 1. Introduction

Antimicrobial resistance (AMR) is widely recognized as a critical and escalating threat to global health systems [1]. Projections suggest that, if current trends persist, AMR-related infections could account for 10 million deaths annually by 2050, underscoring their growing clinical and societal impact [2,3]. A comprehensive assessment of its current burden, spatial distribution, and the predominant pathogen–antimicrobial associations is essential for the development of targeted mitigation strategies [4]. In the absence of effective and coordinated interventions, the ongoing dissemination of resistant strains is likely to exacerbate the severity and mortality of bacterial infections in the near future [5].

To date, over 20,000 species of aromatic and medicinal plants are used in traditional medicine worldwide [6,7]. These plants represent an invaluable source of chemical diversity, providing numerous therapeutic agents employed in both traditional and modern medical systems. They contain a wide range of bioactive compounds, including flavonoids, essential oils, vitamins, saponins, carotenoids, terpenes, polyphenols, and alkaloids, primarily derived from secondary metabolism [8,9,10]. In recent years, growing concerns about the adverse effects of synthetic substances on human health and the environment have increased interest in natural plant-derived products [11]. Among these, herbs, spices, and their essential oils have attracted particular attention due to their multiple biological properties. Essential oils are complex mixtures of volatile metabolites extracted from plant tissues. Their chemical composition can vary depending on several factors, including plant species, plant organ, pedoclimatic conditions, harvest time, physiological stage, and extraction method [12]. These extracts, obtained by distillation or solvent-based methods, exhibit diverse biological activities—including antioxidant, anticancer, anti-inflammatory, and antimicrobial effects—thereby offering promising alternatives to combat the rising threat of antimicrobial resistance [13,14,15,16,17]. Morocco, located at the intersection of the Atlantic Ocean and the Mediterranean Sea in northwestern Africa, is characterized by remarkable climatic diversity. This variability, combined with complex topographic and edaphic factors, generates a wide range of ecological niches, resulting in exceptional floral and faunal richness [18,19,20,21]. *Matricaria pubescens* Desf., belonging to the Asteraceae family, is widely distributed in arid and desert regions of Morocco, as well as in the Saharan areas of Algeria, Tunisia, and Libya [22]. It preferentially grows in clay-sandy habitats, particularly in riverbeds [23]. In traditional medicine, it has been widely utilized for the treatment of various conditions, including rheumatism, gastric ulcers, urinary disorders, scorpion envenomation, dysmenorrhea, dehydration, cough, allergies, and toothache [24,25,26,27]. *M. pubescens* has been reported to exhibit a wide range of pharmacological activities, including anti-inflammatory, antihypertensive, antihyperlipidemic effects, hepatoprotective and nephroprotective properties [28,29,30,31]. Furthermore, extracts of this plant have been shown to inhibit key enzymes associated with type II diabetes and Alzheimer’s disease, highlighting its therapeutic potential [32]. Phytochemically, the species is rich in secondary metabolites such as flavonoids, coumarins, phenols, amides, and sesquiterpenes. Essential oils extracted from *M. pubescens* contain several major compounds, including isochrysanthemic ethyl ester, spathulenol, α-cadinol, geranyl isovalerate, elemicine, herniarin, β-ocimene (Z and E forms), limonene, and myrcene [33,34,35]. Within this context, the present study aimed to characterize, for the first time, the chemical variability of essential oils from two geographically distinct populations of *M. pubescens* from southeastern Morocco and to evaluate their biological potential through antioxidant and antimicrobial assays against a range of microbial strains known for their resistance to multiple standard antibiotics.

## 2. Materials and Methods

### 2.1. Plant Material

*Matricaria pubescens* (Asteraceae) specimens were collected in May 2024 from two locations in the Drâa-Tafilalet region of southeastern Morocco: Errachidia and Zagora. The geographical characteristics of the collection sites are summarized in Table 1.

### 2.2. Essential Oil Extraction

The essential oils were extracted via steam distillation. Approximately 100 g of fresh aerial plant material was evenly distributed on a grid inside the distillation apparatus. Steam generated by a boiler was introduced at the base to pass through the plant material, and the process was maintained for 5 h. The resulting distillate was condensed and collected in a Florentine flask. Following phase separation, the oil fraction was manually isolated, transferred to a brown glass vial, and stored at 4 °C until further analysis [36].

### 2.3. Quantification of Essential Oil Yield

The essential oil yield was expressed as the ratio of the mass of oil recovered to the mass of the plant material used [37] and was calculated according to the following equation:

R = M/M0 × 100(1)

where:

- R: Essential oil yield (%);
- M: Mass of essential oil obtained (g);
- M0: Mass of plant material (g).

### 2.4. Gas Chromatography-Mass Spectrometric (GC/MS) Analysis

The essential oils obtained from the aerial parts of two *M. pubescens* populations were subjected to gas chromatography coupled with mass spectrometry (GC–MS system, TRACE 1300 model, Thermo Fisher Scientific, Waltham, MA, USA). Analyses were performed on a system equipped with a TG-5MS capillary column (30 m × 0.25 mm × 0.25 µm). The injector and transfer line temperatures were maintained at 230 °C and 250 °C, respectively, and ionization was achieved by electron impact at 70 eV. The oven temperature was initially set at 60 °C, then ramped at 3 °C/min to 230 °C and held for 10 min. Each sample (1 µL), previously diluted at a ratio of 1:100 in acetone, was injected under the appropriate operating conditions. Helium served as the carrier gas at a constant flow rate of 1 mL/min. Compound identification was achieved by comparing both the retention indices and mass spectra with those reported in the NIST (National Institute of Standards and Technology) library. Relative quantification of the constituents was performed using the external standard method, based on calibration curves established with reference compounds analyzed under the same chromatographic conditions.

### 2.5. Biological Activities

#### 2.5.1. Antioxidant Activity

The antioxidant potential of the two *M. pubescens* populations was assessed using the DPPH, RPC, and ABTS assays in accordance with established procedures of Von Gadow et al. [38] for DPPH, Oyaizu [39] for RPC, and Li et al. [40] for ABTS. Results were expressed as IC_50_ values, representing the concentration required to inhibit 50% of free radicals. These values were determined graphically using linear regression of inhibition percentages plotted against different concentrations of the tested samples. Butylated hydroxytoluene (BHT) was used as the reference standard (positive control) for each assay due to its well-established antioxidant properties.

#### 2.5.2. Evaluation of Antimicrobial Activity

Microbial Strains

The antimicrobial activity of essential oils from the two *M. pubescens* populations was tested against the following strains: *Staphylococcus aureus* (ATCC 6538), *Bacillus subtilis* subsp. *spizizenii* (ATCC 6633), *Escherichia coli* (ATCC 8739), *Salmonella abony* (NCTC 6017), *Pseudomonas aeruginosa* (ATCC 9027), *Klebsiella pneumoniae*, *Candida albicans* (ATCC 10231), and *Trichophyton rubrum*.

Preparation of Microbial Inoculum

From fresh cultures of the microbial strains, 1–2 well-isolated colonies were picked using a platinum loop and suspended in 10 mL of physiological saline.

After vortexing, bacterial suspensions were standardized to 1–2 × 10^8^ CFU/mL, and fungal suspensions were adjusted to 1–5 × 10^6^ CFU/mL.

Antimicrobial Testing

(a) Disc Diffusion Method

Mueller–Hinton agar was used for bacterial strains, while Sabouraud agar served as the medium for fungal strains. Sterile paper discs (6 mm in diameter) were impregnated with 7 µL of essential oils (10 mg/mL and dilutions of 1/1, 1/10, 1/100, and 1/1000, prepared in 10% DMSO) and placed on pre-inoculated agar plates. Negative controls (10% DMSO) and positive controls (Imipenem 10 µg/disc for bacteria and Fluconazole 25 µg/disc for fungi) were included. Plates were kept at 4 °C for 30 min to allow diffusion of active compounds, then incubated at 37 °C for 24 h for bacteria and 30 °C for 48 h for fungi. Inhibition zone diameters were measured after incubation. All tests were performed in triplicate [41].

(b) Broth Microdilution Method

This approach was applied to determine the minimum inhibitory concentration (MIC) and the minimum fungicidal concentration (MFC) of the tested extracts. A stock solution (10 mg/mL) was prepared in 10% dimethyl sulfoxide (DMSO). An aliquot of 100 µL of this solution was dispensed into the first well of a microtiter plate containing 100 µL of the appropriate culture medium, namely Mueller–Hinton broth (MHB) for bacterial strains or Sabouraud broth (SB) for fungal strains. Two-fold serial dilutions were subsequently performed across the plate to obtain final concentrations ranging from 10 to 0.312 mg/mL. Thereafter, 50 µL of a standardized microbial inoculum was added to each well, resulting in a final volume of 150 µL per well.

The following controls were used:

- Negative control: Culture medium (MHB or SB) + 10% DMSO + microbial suspension.
- Positive control: Culture medium containing either Imipenem (10 µg/mL) for bacteria or Fluconazole (25 µg/mL) for fungi + microbial suspension.
- Neutral control: Culture medium (MHB or SB) + microbial suspension, without extract or antibiotic/antifungal.

The microplates were incubated at 37 °C for 24 h for bacterial strains and at 30 °C for 48 h for fungal strains. Following incubation, 10 µL of a 0.01% (*w*/*v*) resazurin solution was added to each well, and the plates were further incubated for 4–5 h. The minimum inhibitory concentration (MIC) values were subsequently determined. All assays were performed in triplicate in accordance with the method described by Mann and Markham [42].

### 2.6. Statistical Analysis

All experimental data were analyzed using SPSS version 23.0 (IBM, Armonk, NY, USA). One-way ANOVA was applied, and pairwise mean comparisons were performed using Fisher’s LSD test. Statistical significance was defined as *p* < 0.05.

## 3. Results and Discussion

### 3.1. Essential Oil Yield Determination

Essential oils from the two *M. pubescens* populations were extracted by steam distillation under strictly identical conditions. The results (Table 2) revealed notable differences in yield according to geographical origin: the Zagora population showed the lowest yield (0.14%), whereas the Errachidia population exhibited the highest yield (0.23%). The essential oil yields obtained in this study are generally comparable to those reported by previous studies on *M. pubescens* collected in different regions of Algeria. Values ranging from 0.17% (Biskra region) to 0.80% (Béchar region) have been reported by Makhloufi et al. [43] and Bouziane [34]. El Mekhadmi [44] reported an average yield of 0.40 ± 0.27%, with the lowest value (0.06%) recorded in the population cultivated at Ben Neser and the highest (0.92%) observed in the population from El-Menea. This variation in essential oil yield can be attributed to several factors, including the geographical origin of the sample, harvest period, plant organ used, drying duration, and extraction method. Additional factors such as soil type, altitude, exposure, and the plant’s physiological age may also significantly influence essential oil yield [45,46,47].

### 3.2. Chemical Composition of the EOs

GC–MS analysis identified 54 constituents, representing 98.71 ± 0.33% of the total essential oil composition in both *M. pubescens* populations (Table 3). Population EO1 was characterized by isochrysanthemic ethyl ester (32.70%), α-ocimene (17.50%), and (E)-caryophyllene (8.89%), whereas EO2 showed a distinct chemical profile (chemotype) dominated by α-ocimene (19.62%), isochrysanthemic ethyl ester (18.21%), and α-pinene (12.02%). At the chemical class level, monoterpene hydrocarbons were predominant, accounting for 59.62% in EO1 and 53.36% in EO2, followed by sesquiterpene hydrocarbons (15.01% and 18.72%, respectively). Oxygenated monoterpenes, oxygenated sesquiterpenes, phenolic compounds, and other minor constituents occurred in lower proportions in both populations. Overall, the two essential oils exhibited marked qualitative and quantitative variability. Several constituents were detected exclusively in one population, while shared compounds showed substantial differences in relative abundance. These compositional shifts support the classification of the samples into two distinct chemotypes, allowing clear discrimination between the Errachidia and Zagora populations based on their volatile profiles. Chemotype EO1, dominated by isochrysanthemic ethyl ester, is comparable to that reported for populations from Bâcher and Ghardaïa (Algeria) [33,48]. In contrast, chemotype EO2, characterized by α-pinene, closely resembles samples collected from In Amenas and Debdeb (Algeria) [44]. The predominance of monoterpene hydrocarbons observed here agrees with previous reports on *M. pubescens*. Bouziane [34] described essential oil from the Biskra region as rich in oxygenated monoterpenes, with herniarin and spathulenol as major constituents. Similarly, Mekhadmi et al. [41] reported that oils from several populations were largely dominated by monoterpene hydrocarbons (83.75%), particularly Z-β-ocimene (47.41%) and α-pinene (19%). Substantial chemical variability has also been documented within the genus Matricaria. For example, *Matricaria chamomilla* essential oil from Morocco is mainly composed of chamazulene, cis-β-farnesene, and 1,8-cineole [49,50]. In contrast, samples from southern Egypt are dominated by cis-β-farnesene and α-bisabolol oxide A [43]. In addition, *Matricaria recutita* L. from Tehran (Iran) is enriched in trans-trans-farnesol, guaiazulene, and cis-β-farnesene [51]. Overall, essential oil composition is strongly influenced by geographic origin, environmental conditions, the plant organ used, and extraction procedures, which can significantly affect both the qualitative and quantitative profiles of volatile constituents [52,53].

### 3.3. Antioxidant Activity

The antioxidant activity of the two *M. pubescens* essential oils (EO1 and EO2) was expressed as IC_50_ values (Table 4), which are inversely proportional to antioxidant efficacy. Overall, antioxidant capacity differed between the two samples depending on the assay employed. Across all tests, EO2 consistently exhibited lower IC_50_ values than EO1, indicating stronger antioxidant activity. Nevertheless, both essential oils remained less active than the reference standard BHT, which showed the lowest IC_50_ values. In the DPPH radical-scavenging assay, EO2 showed greater activity (IC_50_ = 0.95 ± 0.10 mg/mL) than EO1 (IC_50_ = 1.34 ± 0.04 mg/mL), reflecting a higher free radical neutralization capacity. A similar trend was observed in the reducing power assay, which evaluates the ability of antioxidants to reduce Fe^3+^ to Fe^2+^ via electron transfer. In this test, IC_50_ values were 1.23 ± 0.05 mg/mL for EO2 and 1.91 ± 0.09 mg/mL for EO1, further confirming the superior antioxidant potential of EO2. Likewise, the ABTS assay, based on the reduction of the ABTS^+^·cation radical, indicated stronger activity for EO2 (IC_50_ = 0.76 ± 0.12 mg/mL) compared with EO1 (IC_50_ = 1.05 ± 0.09 mg/mL). These findings are consistent with those reported by Mekhadmi et al. [37], who assessed the radical-scavenging activity of 14 *M. pubescens* essential oil samples and reported IC_50_ values ranging from 0.762 to 36.26 mg/mL, highlighting the notable antioxidant potential of this species. The antioxidant activity of essential oils is strongly dependent on their chemical composition. Harkat et al. [54] emphasized that both the nature and concentration of major constituents play a key role in determining antioxidant efficacy. However, several studies have suggested that synergistic interactions between major and minor compounds can substantially modulate the overall activity of essential oils [55,56,57]. In addition, essential oils enriched in oxygenated compounds have frequently been reported to exhibit antioxidant activity comparable to, or even greater than, oils dominated by hydrocarbon terpenes [58,59,60]. Phenolic compounds, in particular, are well known for their strong radical-scavenging capacity due to the presence of hydroxyl groups, which facilitate electron or hydrogen atom transfer reactions [61,62]. Finally, unsaturated terpenes such as limonene and α-pinene may also contribute to antioxidant activity, especially through synergistic effects that enhance the efficacy of complex mixtures [63,64].

### 3.4. Antibacterial Activity

The results presented in Table 5 show that the essential oils of *M. pubescens* exhibited marked antimicrobial activity against most of the tested strains. Overall, oil EO2 demonstrated inhibitory effects against all microorganisms, although sensitivity varied among strains. In contrast, EO1 showed no activity against *Salmonella abony* and *Pseudomonas aeruginosa*. A clear dose-dependent response was observed for both oils, as increasing dilution led to a progressive reduction in inhibition zone diameters in susceptible strains. In some cases, activity was completely lost at a dilution of 1/10, and at 1/100 or 1/1000 for all tested strains. For EO1, inhibition zones ranged from 10 to 22 mm. *Staphylococcus aureus* and *Bacillus subtilis* were the most sensitive strains, with inhibition zones of 22 ± 1.0 mm and 16 ± 0.8 mm, respectively. The lowest susceptibility was observed for *Klebsiella pneumoniae* and *Trichophyton rubrum*, with inhibition zones of 14 ± 0.8 mm and 14 ± 1.1 mm, respectively. For EO2, inhibition zones ranged from 10 to 25 mm. *Bacillus subtilis* and *Escherichia coli* showed the highest sensitivity, with inhibition zones of 25 ± 1.2 mm and 23 ± 1.3 mm, respectively. In contrast, *Pseudomonas aeruginosa* and *Salmonella abony* were the least sensitive, with inhibition zones of 12 ± 0.8 mm and 10 ± 1.0 mm, respectively. Overall, the results indicate that *M. pubescens* essential oils were active against both Gram-positive and Gram-negative bacteria, with a noticeable difference in susceptibility between the two bacterial groups. Regarding fungi, both oils exhibited comparable antifungal activity, although slight variations in inhibition zone diameters were observed.

### 3.5. MIC and MFC of M. pubescens Essential Oil

The MIC and MFC values of the two *M. pubescens* essential oils (EO1 and EO2) were determined using the microdilution method. Table 6 summarizes the MIC and MFC values obtained against the tested bacterial and fungal strains. Overall, both oils exhibited antimicrobial activity, although efficacy varied depending on the microorganism. Oil EO2 generally showed slightly higher activity than EO1, particularly against *Staphylococcus aureus* (MIC = 0.612 mg/mL for EO2 vs. 2.5 mg/mL for EO1) and *Klebsiella pneumoniae* (MIC = 1.25 mg/mL for EO2 vs. 2.5 mg/mL for EO1). In addition, EO2 displayed stronger antifungal activity against *Trichophyton rubrum* (MFC = 1.25 mg/mL) compared with EO1 (MFC = 2.5 mg/mL), whereas *Candida albicans* showed the same MFC value (2.5 mg/mL) for both oils. Among Gram-positive bacteria, *Bacillus subtilis* subsp. *spizizenii* exhibited equal sensitivity to both oils, with a MIC of 0.612 mg/mL, indicating a strong inhibitory effect of *M. pubescens* essential oils against this strain. In contrast, Gram-negative bacteria such as *Pseudomonas aeruginosa* and *Escherichia coli* were less susceptible, with relatively higher MIC values ranging from 1.25 to 2.5 mg/mL for both EO1 and EO2. Overall, these results suggest that *M. pubescens* essential oils possess promising antimicrobial potential, with stronger activity against Gram-positive bacteria and pathogenic fungi. Differences in antimicrobial efficacy between EO1 and EO2 may be closely linked to variations in their chemical composition, particularly the relative abundance of bioactive constituents, as well as to the intrinsic characteristics of the target microorganisms. These findings are consistent with those reported by Mekhadmi et al. [41], who observed notable resistance of Pseudomonas aeruginosa to *M. pubescens* essential oils, while *Proteus mirabilis* and *Staphylococcus aureus* were more susceptible. Similarly, Makhloufi et al. [43] reported significant antibacterial activity of *M. pubescens* oils against both Gram-positive and Gram-negative strains. In contrast, Bouziane et al. [34] described marked resistance of several bacterial strains to *M. pubescens* essential oils collected from the Ghardaïa region, suggesting that geographic and ecological factors may strongly influence oil composition and, consequently, biological activity. Comparable variability has also been reported in other species of the genus Matricaria. Mekonnen et al. [65] found that *Matricaria chamomilla* essential oil exhibited no antibacterial activity against *Staphylococcus aureus*, *Salmonella typhi*, *Escherichia coli*, and *Pseudomonas aeruginosa*. Conversely, Ferhat et al. [66] reported antimicrobial effects of *M. chamomilla* oil against *Staphylococcus aureus*, *Bacillus cereus*, *Bacillus subtilis*, *Proteus sp.*, and *Shigella shiga*, while *P. aeruginosa* remained resistant. Several studies have shown that essential oils, particularly those rich in monoterpene hydrocarbons and oxygenated terpenes, can exert strong antimicrobial effects mainly through disruption of microbial membrane integrity [67,68]. In this regard, Han et al. [69] demonstrated that limonene induces severe structural damage to the cell wall and plasma membrane of *Listeria monocytogenes*, leading to increased electrical conductivity and leakage of intracellular macromolecules such as proteins and nucleic acids. Microscopic observations confirmed cell lysis, supporting the conclusion that limonene’s antimicrobial activity is primarily related to membrane destabilization. Similarly, essential oils containing monoterpenes such as α-pinene, β-pinene, terpinen-4-ol, and α-terpineol, as well as sesquiterpenes such as β-caryophyllene oxide, have been reported to display pronounced antibacterial and antifungal activities [70,71]. These compounds mainly act by altering membrane permeability, resulting in rapid cell damage and growth inhibition. Moreover, terpenes may exert multitarget effects in Escherichia coli, including disruption of cytoplasmic membrane integrity, inhibition of respiratory chain enzymes, and interference with ion transport systems, ultimately leading to cell death [67,72]. In line with this, Mekhadmi et al. [41] reported that *M. pubescens* essential oils are typically dominated by monoterpenes and sesquiterpene hydrocarbons, with major constituents including (Z)-β-ocimene, α-pinene, β-bulnesene, allo-ocimene, and 1-phenyl-penta-2,4-diene, which may contribute to the antimicrobial activity of this species. Importantly, antimicrobial efficacy depends not only on the chemical profile of essential oils but also on the Gram status of the target microorganisms. Indeed, Gram-positive bacteria generally exhibit higher sensitivity to essential oils than Gram-negative bacteria, whose increased resistance is mainly attributed to the presence of a complex outer membrane that limits the penetration of hydrophobic compounds [73,74]. The findings indicate that Gram-positive bacteria were more susceptible to *M. pubescens* essential oils than Gram-negative strains, likely due to structural differences in their cell walls. This selective antimicrobial activity highlights the potential of *M. pubescens* as a source of natural compounds capable of combating multidrug-resistant pathogens and supports its traditional use, providing a basis for future therapeutic investigations.

## 4. Conclusions

This study demonstrates that *M. pubescens* from southeastern Morocco is a valuable source of bioactive compounds, with significant antioxidant and antimicrobial potential. Distinct chemical profiles were observed between populations from Errachidia and Zagora, indicating the presence of different chemotypes. The strong free radical-scavenging activity and selective antimicrobial effects against key pathogens highlight the species’ pharmacological relevance. These findings provide a scientific basis for its traditional medicinal use and support further investigations into its therapeutic applications.

## Figures and Tables

**Table 1:** Geographical characteristics of the two sampling sites.

Site	Latitude	Longitude	Altitude (m)
Errachidia	31.938976	−3.615810	938
Zagora	30.250028	−5.679229	654

**Table 2:** Essential Oil Yield (%) of *M. pubescens*.

Oils	Yield (%)
EO1	0.23
EO2	0.14

EO1: essential oil from the Errachidia site; EO2: essential oil from the Zagora site.

**Table 3:** Chemical composition of the essential oils of *M. pubescens*.

Compounds	RI	Content %	
		EO1	EO2
α-Pinene	926	6.88	12.02
Camphene	947	0.1	0.15
Sabinene	972	0.22	0.33
α-Myrcene	990	0.3	0.21
p-Cymene	1014	0.08	0.3
Limonene	1024	0.45	1.03
α-Ocimene	1031	17.52	19.62
β-Ocimene (Z)	1047	0.86	1.49
Linalool	1084	0.00	0.1
α-Pinene oxide	1094	0.5	0.00
3-Oxatricyclo[4.1.1.0(2,4)]octane, 2,7,7-trimethyl	1101	0.14	0.3
3-Methyl-2-(2-methyl-2-butenyl)-furan	1106	0.18	0.08
Thujone	1110	0.21	0.43
α-Thujone	1113	0.1	0.3
Cis-3-Hexenyl isovalerate	1115	0.05	0.00
Cis-Epoxy-Ocimene	1128	0.13	0.22
Camphor	1142	0.00	0.19
cis-α-Terpinol	1163	0.03	0.05
Cis-Verbenol	1148	0.11	0.09
Linalyl acetate	1252	0.00	0.15
Bornyl acetate	1272	0.18	0.43
(-)-Myrtenyl acetate	1324	0.00	0.1
Copaene	1366	0.55	0.3
(-)-α-Bourbonene	1380	0.08	0.00
ζ-Elemene	1401	0.00	1.82
Methyleugenol	1409	0.05	0.6
β-Farnesene (E)	1416	2.12	1.35
(E)-Caryophyllene	1427	8.89	7.87
Germacrene D	1443	1.97	3.89
α-Humulene	1457	0.74	1.12
Trans-α-Ionone	1461	0.37	0.00
(S,E)-2,5-Dimethyl-4-vinylhexa-2,5-dien-1-yl acetate	1481	0.11	0.00
α-Bisabolene	1507	0.07	1.27
β-Germacrene-1-ol	1515	0.00	0.36
(1R,7S,E)-7-Isopropyl-4,10-dimethylenecyclodec-5-enol	1633	0.00	0.2
Elemicin	1648	2.79	2.23
(-)-Spathulenol	1558	0.95	0.65
α-Cadinol	1561	2.01	2.68
Isoaromadendrene epoxide	1567	0.72	0.43
Corymbolone	1571	0.00	0.32
Caryophyllene oxide	1579	7.21	11.33
Trans-Z-α-Bisabolene epoxide	1590	0.00	0.15
Geranyl isovalerate	1593	0.00	0.75
Aristolene epoxide	1597	0.46	0.00
α-Humulene epoxide II	1611	0.92	0.47
Junenol	1617	0.51	0.00
1-Acenaphthenol	1637	0.00	0.15
γ-Muurolene	1657	0.66	0.93
α-Bisabolol	1670	0.9	0.49
Dodecyl acrylate	1679	0.43	0.75
Isochrysanthemic ethyl ester	1695	32.71	18.21
Hanphyllin	1704	3.7	2.52
Guaiazulene	1770	0.00	0.17
Neophytadiene	1829	1.54	0.44
Total compounds		42	47
Total content %		98.38	99.04
Monoterpene hydrocarbons		59.62	53.36
Oxygenated monoterpenes		4.89	5.71
Sesquiterpene hydrocarbons		13.68	17.08
Oxygenated sesquiterpenes		15.01	18.72
Phenolic compounds		2.84	2.83
Others		2.34	1.34
Unidentified compounds		1.62%	0.96%

RI: Retention index experimentally determined against a series of n-alkanes (C7–C40); EO1: essential oil from the Errachidia site; EO2: essential oil from the Zagora site.

**Table 4:** Comparative Antioxidant Activity of EO1 and EO2 Essential Oils of *M. pubescens*.

Oils	DPPH IC_50_ (mg/mL)	RPC IC_50_ (mg/mL)	ABTS IC_50_ (mg/mL)
EO1	1.34 ± 0.04 ^c^	1.91 ± 0.09 ^c^	1.05 ± 0.09 ^c^
EO2	0.95 ± 0.10 ^b^	1.23 ± 0.05 ^b^	0.76 ± 0.12 ^b^
BHT *	0.04 × 10^−3^ ± 0.001 ^a^	2.41 × 10^−3^ ± 0.007 ^a^	0.21 × 10^−3^ ± 0.002 ^a^

EO1: essential oil from the Errachidia site; EO2: essential oil from the Zagora site; * BHT: Butylated hydroxytoluene. All data are presented as mean ± standard deviation (SD) from three independent replicates (*n* = 3). Values followed by different lowercase letters are significantly different according to LSD test (*p* ≤ 0.05).

**Table 5:** Antimicrobial activity of *M. pubescens* essential oils (inhibition zone in mm).

Strains EOs	*B.* *subtilis*	*S.* *aureus*	*S.* *abony*	*E.* *coli*	*P. aeruginosa*	*K. pneumoniae*	*C. albicans*	*T. rubrum*
EO1-1/1	16 ± 0.8 ^d^	22 ± 0.7 ^b^	6 ± 0.0 ^d^	15 ± 1.3 ^c^	6 ± 0.0 ^c^	14 ± 0.8 ^c^	16 ± 0.5 ^c^	14 ± 1.1 ^c^
EO1-1/10	10 ± 1.1 ^e^	14 ± 1.2 ^d^	6 ± 0.0 ^d^	8 ± 0.5 ^e^	6 ± 0.0 ^c^	11 ± 0.3 ^d^	8 ± 0.7 ^d^	9 ± 0.4 ^b^
EO1-1/100	6 ± 0.0 ^f^	6 ± 0.0 ^e^	6 ± 0.0 ^d^	6 ± 0.0 ^f^	6 ± 0.0 ^c^	6 ± 0.0 ^e^	6 ± 0.0 ^e^	6 ± 0.0 ^e^
EO1-1/1000	6 ± 0.0 ^f^	6 ± 0.0 ^e^	6 ± 0.0 ^d^	6 ± 0.0 ^f^	6 ± 0.0 ^c^	6 ± 0.0 ^e^	6 ± 0.0 ^e^	6 ± 0.0 ^e^
EO2-1/1	25 ± 1.2 ^b^	20 ± 1.0 ^c^	10 ± 0.4 ^b^	23 ± 1.3 ^b^	12 ± 1.0 ^b^	21 ± 1.6 ^b^	18 ± 1.0 ^b^	17 ± 1.4 ^b^
EO2-1/10	18 ± 0.9 ^c^	15 ± 0.3 ^d^	8 ± 0.6 ^c^	12 ± 1.0 ^d^	6 ± 0.0 ^c^	14 ± 1.0 ^c^	8 ± 0.5 ^d^	9 ± 1.0 ^d^
EO2-1/100	6 ± 0.0 ^f^	6 ± 0.0 ^e^	6 ± 0.0 ^d^	6 ± 0.0 ^f^	6 ± 0.0 ^c^	6 ± 0.0 ^e^	6 ± 0.0 ^e^	6 ± 0.0 ^e^
EO2-1/1000	6 ± 0.0 ^f^	6 ± 0.0 ^e^	6 ± 0.0 ^d^	6 ± 0.0 ^f^	6 ± 0.0 ^c^	6 ± 0.0 ^e^	6 ± 0.0 ^e^	6 ± 0.0 ^e^
Imipenem	40 ± 0.12 ^a^	42 ± 0.56 ^a^	38 ± 0.17 ^a^	36 ± 0.30 ^a^	24 ± 0.15 ^a^	25 ± 0.10 ^a^	ND	ND
Fluconazole	ND	ND	ND	ND	ND	ND	28 ± 0.25 ^a^	18 ± 0.28 ^a^

EO1: Essential oil of *M. pubescens* from the Errachidia site; EO2: Essential oil of *M. pubescens* from the Zagora site; ND: Not Determined. All data are presented as mean ± standard deviation (SD) from three independent replicates (n = 3). Values followed by different lowercase letters are significantly different according to LSD test (*p* ≤ 0.05).

**Table 6:** MIC and MFC of *M. pubescens* Essential Oils Against Bacterial and Fungal Strains (mg/mL).

	Bacterial Strains (MIC)						Fungal Strains (MFC)	
Oils	*S. aureus*	*B. subtilis*	*S. abony*	*E. coli*	*P. aeruginosa*	*K. pneumonie*	*C. albicans*	*T. rubrum*
EO1	2.5	0.612	2.5	1.25	2.5	2.5	2.5	2.5
EO2	1.25	0.612	2.5	1.25	2.5	1.25	2.5	1.25

EO1: Essential oil of *M. pubescens* from the Errachidia site; EO2: Essential oil of *M. pubescens* from the Zagora site.

## Data Availability

The original contributions presented in this study are included in the article. Further inquiries can be directed to the corresponding author(s).
